# Understanding Nanocalcification: A Role Suggested for Crystal Ghosts

**DOI:** 10.3390/md12074231

**Published:** 2014-07-23

**Authors:** Ermanno Bonucci

**Affiliations:** La Sapienza University, Policlinico Umberto I, Viale Regina Elena 324, Rome 00161, Italy; E-Mail: ermanno.bonucci@uniroma1.it; Tel.: +39-064-957-685

**Keywords:** biomineralization, bone, calcification, crystal ghosts, crystallites, electron microscopy, organic-inorganic relationships, templates

## Abstract

The present survey deals with the initial stage of the calcification process in bone and other hard tissues, with special reference to the organic-inorganic relationship and the transformation that the early inorganic particles undergo as the process moves towards completion. Electron microscope studies clearly exclude the possibility that these particles might be crystalline structures, as often believed, by showing that they are, instead, organic-inorganic hybrids, each comprising a filamentous organic component (the crystal ghost) made up of acidic proteins. The hypothesis is suggested that the crystal ghosts bind and stabilize amorphous calcium phosphate and that their subsequent degradation allows the calcium phosphate, once released, to acquire a hydroxyapatite, crystal-like organization. A conclusive view of the mechanism of biological calcification cannot yet be proposed; even so, however, the role of crystal ghosts as a template of the structures usually called “crystallites” is a concept that has gathered increasing support and can no longer be disregarded.

## 1. Introduction

In the course of 1926, using X-ray diffraction, De Jong [1] showed that the inorganic fraction of bone consists of very small particles, whose diffractograms are similar to those of poorly crystalline carbonated apatite. These findings were confirmed a few years later by Roseberry *et al.* [2] and subsequently by a number of investigators using the same or different physical techniques in a number of hard tissues. In addition, studies with the polarizing microscope suggested that, in accordance with Wiener’s law, the inorganic particles in bone are structured like rods [3], and the electron microscope confirmed that they are needle- or platelet-like nanostructures [4,5]. Similar results were drawn from studies on other calcified tissues of both terrestrial and marine vertebrates, such as epiphyseal cartilage, dentine and enamel, and were extended to invertebrates, whose shells and spicules consist of aragonite and calcite plates (reviewed by [6]). These and other observations led to the conclusion that the inorganic structures of vertebrate hard tissues are rod-, needle- or platelet-like nanoparticles consisting of polycrystalline hydroxyapatite; they therefore came to be called “crystals” or “crystallites” and were thought to respond to the rules of mineralogy, although, as stressed by Arnott and Pautard [7], there were no proofs that, in developing bone, any portion of the area where they occur is specifically crystalline.

The conviction that the inorganic structures of bone are “crystals” led to attempts to explain their formation and properties according to the rules of mineralogy. On the other hand, there has been an increasing awareness that the biomineralization process occurs in the context of an organic matrix and that this plays a conditioning role in mineralization, by promoting or by inhibiting the deposition of the inorganic substance. A number of theories based on prevalently mineralogical or on prevalently biological concepts have therefore been put forward; in neither case has any definitive explanation of the mechanism of biomineralization emerged. As a result, the whole topic is still widely debated.

The main obstacle to finding a definitive solution to the controversial issue of the mechanism of biomineralization seems to be the widespread, deeply rooted conviction that, first, the inorganic substance has, from the outset, a crystalline organization, which then persists unchanged until the tissue is eventually reabsorbed, whereas the actual evidence is that the earliest mineral particles are non-crystalline; second, that the bone “crystals” (the terms “crystal” and “crystallite”, as well as “mineralization” and “biomineralization” are retained here from force of habit) are considered stable, permanent structures, when they actually undergo deep, although poorly known, changes during their lifespan. These are not only the well-known changes in crystal chemistry that occur during the aging of animals, as already described for bone by Posner *et al.* [8] in 1965 and then confirmed by X-ray diffraction in the same type of tissue [9,10,11,12], as well as by nuclear magnetic resonance in bone and enamel [13], but also other structural modifications that take place as the earliest inorganic particles evolve into the definitive needle-shaped, crystal-like structures. These changes imply the acquisition of a more apatite-like configuration and a higher degree of crystallinity [9,12,14], which, in turn, imply an increase in crystallite size, an attenuation in lattice imperfections, or both [14]. They take the form of a sort of crystal “maturation” that is usually overlooked, although it cannot be ignored without damaging our understanding of the mechanism of calcification.

### 1.1. “Maturation” of Crystals

Morphological studies carried out on both marine and terrestrial organisms have shown that the earliest inorganic particles are non-crystalline and that they undergo a sort of maturation that gradually leads to their becoming hydroxyapatite-like structures. As early as 1977, X-ray studies on oriented bone sections by Wheeler and Lewis [15] had shown that bone apatite has a paracrystalline structure (*i.e.*, no long range order). Electron diffraction studies on different types of hard tissue (bone, dentine, enamel) confirmed that the crystallites that are formed at an early stage have a paracrystalline character comparable to that of biopolymers and that the lattice fluctuations decrease with age and maturation, so allowing the acquisition of a typical crystalline organization [16]. In dentine, the fluctuation of the lattice plane distances in the c-axis direction decreases in proceeding from the region near the dentine/predentine border to the dentine/enamel border [17]. In line with these observations, Landis and Glimcher [18] reported that no electron diffraction pattern of a specific calcium phosphate solid phase is generated from the early mineral deposits of newly synthesized bone, whereas the more heavily calcified, older regions of the bone show the reflections and characteristics of poorly crystalline hydroxyapatite. These results are in agreement with the finding that the Ca/P molar ratio changes with the age of the crystals: Wergedal and Baylink [19] found that the earliest mineral deposits (*i.e.*, calcification nodules) in osteoid tissue have a mean Ca/P ratio value of 1.35, which increases to 1.60 in the fully-calcified areas; in the same areas, Landis and Glimcher [18] reported ratio ranges of 1.60–1.70 and 1.81–1.97, respectively.

An evident example of “crystal maturation” is given by enamel formation (reviewed by Nanci, [20]). The earliest enamel crystals are thin, very long, filament- and ribbon-like structures, which reveal a poor hydroxyapatite electron diffraction and whose electron probe analysis shows a low Ca/P molar ratio (mean value: 1.24 [21]). It is only through a process of “maturation” that enamel crystals acquire their typical hexagonal shape, a Ca/P molar ratio of about 1.40 and give hydroxyapatite electron diffractograms. Several reports pointed to the early enamel mineral as being amorphous calcium phosphate (ACP) that later fuses to become hydroxyapatite [22,23,24], and the same results were reported by Mahamid *et al.* [25] in bones of zebrafish. In addition, Beniash *et al.* [22] made the interesting observation that the size, shape and spatial organization of the early, non-crystalline mineral particles are essentially the same as those of the older crystals; they suggested that the mineral morphology and organization are determined prior to the formation of definitive crystalline structures. This topic was considered also by Simmer *et al.* [24], who agreed that the early enamel consists of non-crystalline ACP and that the mineral in the early enamel ribbons is not yet crystalline and has no shape of its own.

The existence of a phase of maturation, which leads to the gradual development of apatite-like crystals, implies that studies on the mechanism of biomineralization cannot be carried out on the bulk of mature hard tissue, but must be concentrated on the inorganic structures that are formed early and on their evolution.

### 1.2. Early Inorganic Structures

**Figure 1:**
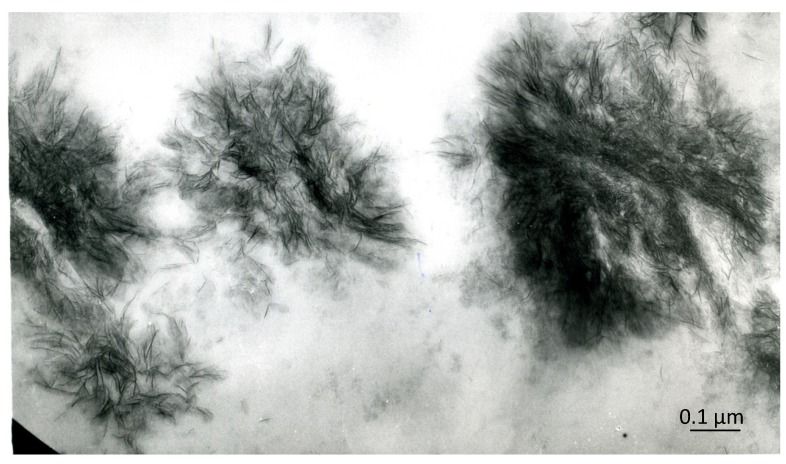
An area of early calcification in bone: the calcification nodules consist of roundish aggregates of filament-like, intrinsically electron-dense structures. Unstained, ×90,000. Scale bar: 0.9 mm = 0.1 µm.

**Figure 2:**
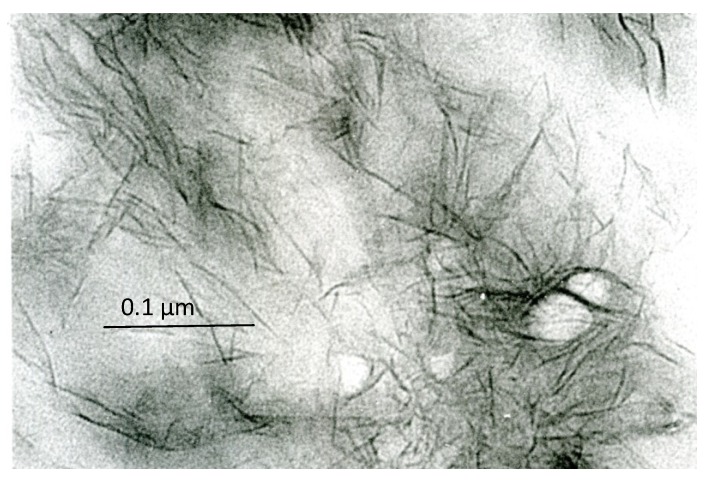
Detail of the inorganic structures that are components of the calcification nodules in bone; note that they have a filament-like appearance. Unstained, ×220,000. Scale bar: 22 mm = 0.1 µm.

The early inorganic structures of vertebrate hard tissues show various aggregation states that can be recognized under the electron microscope: whether in bone, dentin or cartilage, the early recognizable mineralized areas correspond to the so-called “calcification nodules”, which are small, roundish aggregates of filament-like crystallites (Figure 1). In bone, especially compact secondary bone, the inorganic substance shows, in addition, a unique relationship with the periodic banding of the collagen fibrils, whose pattern is reinforced, leading to the ultrastructural picture known as “mineral substance in bands”. In enamel, the early ribbon- and filament-like crystals are oriented almost perpendicularly to the ameloblast plasma membrane and are organized into rod and inter-rod structures. In spite of the different arrangements that are found in different tissues, at the outset, the single crystal units are all rather similar: they are long (or very long, in the case of enamel), thin nanostructures (thickness ranging from 1.0 to 7.7 nm in bone and around 1.5 nm in immature enamel [6]) that are usually compared to needles or rods, or threads and ribbons in enamel, but should, rather, be considered filamentous structures, because of their frequently bent and winding appearance (Figure 2). Only with maturation do they increase in size, acquire a more definite pattern, similar to rigid, inorganic structures, and, as discussed above, give the diffraction pattern of hydroxyapatite. They develop in an organic matrix and, therefore, maintain a close relationship with the organic components.

### 1.3. Organic-Inorganic Relationships

A number of investigations have been carried out in marine and terrestrial organisms to establish the location of the crystals in bone and their relationships with organic structures. The excessive importance attached to collagen fibrils as being responsible for the heterogeneous nucleation of bone crystals and the finding of the “mineral substance in bands” that reinforces the collagen period favored the idea that the bone crystals are nucleated in the hole zone of the collagen fibrils and that they therefore come to be located within the fibrils [26]. It is now clear that the inorganic substance in bone is organized in two different, relatively independent patterns, corresponding in the first case to the “mineral substance in bands”, which is actually contained in the hole zones of the collagen fibrils, and in the second to the filament-like crystals, which are located in the extrafibrillar space [27,28]. This double organization is recognizable in bone, but is hard to identify in dentin, where filament-like crystals predominate; it is completely absent in cartilage and enamel, where only filament-like crystals are recognizable.

The mineral substance in bands and the filament-like crystals may basically be formed through the same process, the differences in mineral organization being exclusively due to the structure (collagenous or not) of the matrix (discussed by Bonucci [28]). The crystals located in the extrafibrillar space are obviously in contact with non-collagenous components of the organic matrix; the recognition of the ultrastructural relationships between organic and inorganic components is, however, a demanding task, not only because of the heterogeneity of the former, but also, and primarily, because of the masking effect of the latter. The inorganic substance is, in fact, electron-dense, and this fact makes it difficult (or impossible) to distinguish it from the organic structures with which it is associated. The practical consequence is that the electron microscope study of the organic components of the calcified matrix requires their unmasking by decalcification.

### 1.4. Decalcification Procedures

The removal of the inorganic substance by decalcification is not without consequences on the components of the organic matrix and may be responsible for a number of artifacts. Two methods can be used to avoid them or to keep them to a minimum and to preserve the ultrastructural morphology of the decalcified tissue: the PEDS (post-embedding decalcification and staining) method and the CDS (cationic dye stabilization) method.

The PEDS method implies that the decalcification is carried out after the tissue has been embedded in a resin, either by flotation of the ultrathin sections on, or the soaking of whole embedded specimens in, the decalcified solution, followed by “staining” with a heavy metal [29,30]. In both cases, the embedding resin stabilizes the organic components, which are retained unaltered in the sections in spite of the complete removal of inorganic substance (discussed by [29]). The CDS method is based on the stabilization of anionic molecules by cationic dyes (Alcian blue, acridine orange, cupromeronic blue, *etc*.) that are added to the fixative solutions [31]. The PEDS method gives the best results: the ultrastructure of the sections decalcified and stained with this method is practically indistinguishable from that of a non-decalcified section.

**Figure 3:**
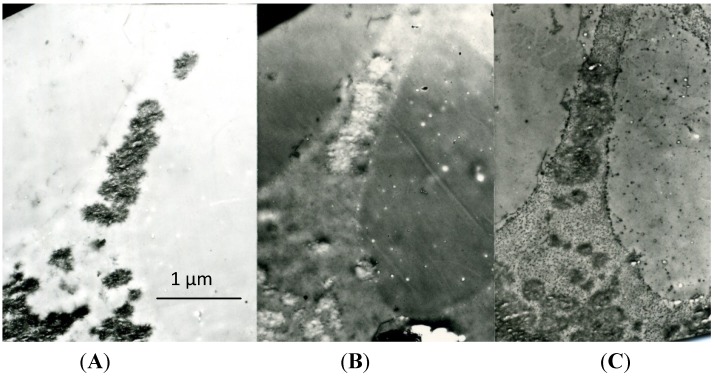
A series of three sections of the same area of epiphyseal cartilage, showing: (**A**) a zone of early calcification (untreated; the calcified matrix is electron-dense); (**B**) a corresponding area after decalcification (decalcified with formic acid and unstained; the previously calcified area is electron-transparent); and (**C**) another corresponding area after decalcification and staining (decalcified with formic acid and stained with uranium and lead; the previously calcified area is electron-dense; compare with Figure 3A). Post-embedding decalcification and staining (PEDS) method, ×16,000. Scale bar: 16 mm = 1 µm.

**Figure 4:**
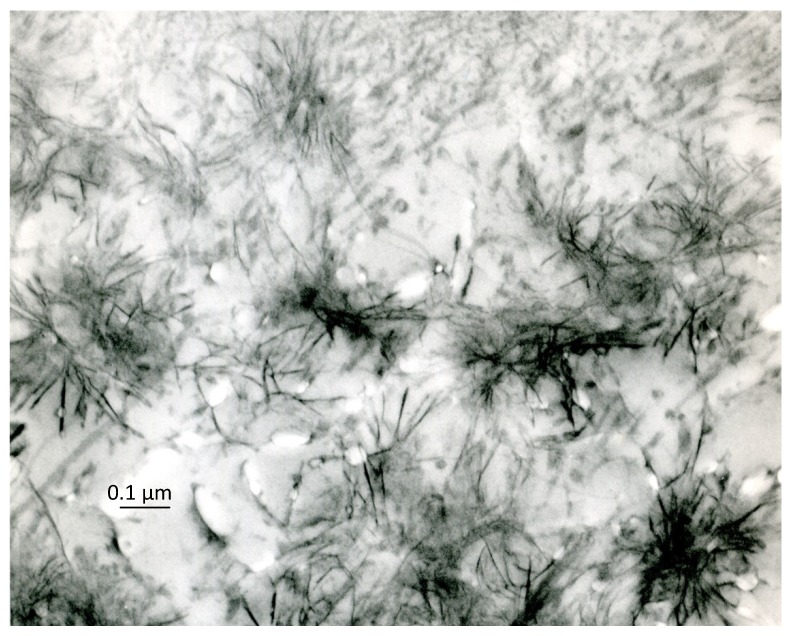
Area of early cartilage calcification after treatment with the PEDS method: the calcification nodules are replaced by similar aggregates of crystal ghosts. Decalcified with formic acid and stained with uranium and lead, ×80,000. Scale bar: 8 mm = 0.1 µm.

Surprisingly, this similarity also applies to the areas of initial calcification: in spite of the decalcification procedure, the previously calcified areas (which appear as empty areas after decalcification alone, *i.e.*, after the first PEDS step) appear electron-dense after the whole PEDS procedure (decalcification and staining; Figure 3). This finding was initially interpreted as being due to the persistence of inorganic crystals, because of the failure of the decalcification process; it is actually due to the deep staining of decalcified, crystal-like, organic structures that were previously masked by the inorganic substance (Figure 4). This result is common to the calcification nodules of bone, dentin, cartilage and, again, to the early, immature enamel, but is particularly conspicuous in calcifying cartilage: the aggregates of filament-like inorganic crystals are replaced by aggregates of filament-like organic structures (crystal ghosts [29]). In contrast, the fully-calcified matrix appears faintly stained and has an amorphous appearance.

### 1.5. Crystal Ghosts

**Figure 5:**
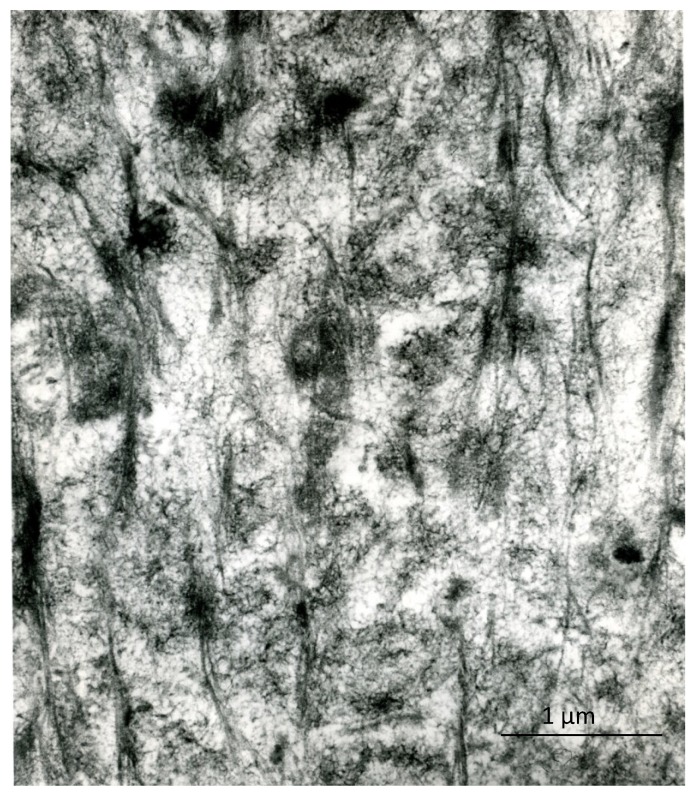
Area of early cartilage calcification after treatment with the cationic dye stabilization (CDS) method: the calcification nodules are replaced by similar aggregates of filament-like structures resembling crystal ghosts. Glutaraldehyde-acridine orange fixation, EDTA decalcification, ruthenium red staining, ×24,000. Scale bar: 24 mm = 1 µm.

Crystal ghosts are organic, filament-like structures that become manifest under the electron microscope when the early calcification areas (calcification nodules) are treated with the PEDS method, *i.e.*, are decalcified after embedding and are then “stained” with uranyl acetate and lead citrate (Figure 4), phosphotungstic acid or other heavy metals (reviewed by [32]). Their name derives from their only becoming manifest when the inorganic crystals are dissolved by decalcification and, above all, from their close morphological similarity with untreated crystals. Structures analogous to crystal ghosts are shown by the CDS method (Figure 5).

Most of the studies on the relationship between the organic and inorganic components during the early stages of calcification have been carried out in calcifying cartilage (reviewed by [33]); however, crystal ghosts have also been recognized in bone [34], dentin [35,36] and enamel [37,38,39,40,41], as well as in non-skeletal tissues [42,43]. The crystal ghosts of the cartilage, which can be considered paradigms of the crystal ghosts in all other hard tissues, are thin (mean: 9 nm), straight or, more often, irregularly wavy structures of variable length; those of early enamel differ above all in their length, which is hard to measure, but in any case considerable (reviewed by [6]). The crystal ghosts have no intrinsic electron-density and become recognizable under the electron microscope only after their staining with heavy metals. Before further considering these structures and in view of the role that they may play in the mineralization process, it is mandatory to exclude any possible artifact that might affect their nature.

First, the similarity of crystal ghosts with untreated crystals is so close, that it might be thought that they are just residual crystals left in the sections, because of incomplete decalcification. This possibility can easily be excluded: the decalcification of ultrathin sections is easy and rapid, even by simple flotation of sections on distilled water [44]; moreover, direct electron microscope examination (Figure 3B) and the electron diffraction of the decalcified areas [37] both confirm the complete dissolution of the inorganic substance.

Second, one possibility that has been put forward is that crystal ghosts may be produced by stain penetration in holes left in the section when crystallites have been extracted [45]. This hypothesis is in conflict with the observation that crystal ghosts are recognizable in sections that were re-embedded after decalcification, so much so, that every space produced and left by decalcification should have been occluded. Moreover, structures similar to crystal ghosts can be demonstrated using the CDS method in which, as reported above, decalcification occurs before embedding.

Third, adsorption of organic material on the crystal surface during the fixation procedure has been considered a possible cause of the formation of crystal ghosts. However, the thickness of the crystal ghosts in cartilage is approximately the same as that of untreated crystals, whereas it would inevitably be greater if they were located around the latter; moreover, on accepting the envelope hypothesis, their cross-section should display them as rings, whereas the image that appears shows them as small dots.

Fourth, the concept that crystal ghosts are organic components of crystals has been considered to be inconsistent, because crystals cannot accommodate proteins, and these are not compatible with the crystal structure [46,47]. This view derives from the deep-rooted opinion that the inorganic structures of hard tissues are true crystals and that, as such, they must be strictly adherent to the laws of mineralogy. As discussed above, the nanostructures that give rise to calcification nodules and that are called “crystals” are not true crystals, do not give crystalline electron diffractograms and only become crystalline with aging and maturation; it should also be noted that they have these properties just because of their link with crystal ghosts. It must be added, anyway, that the location of organic material in the crystals is not necessarily in contrast with their organization: intracrystalline organic material has been reported several times in biological hard tissues, and it can even strengthen the mechanical properties of crystals [48,49,50,51,52].

The considerations set out above point to crystal ghosts as being true organic components of early crystals; histochemical investigations confirm their organic nature. The crystal ghosts of cartilage are, in fact, stained by acidic phosphotungstic acid, periodic acid-silver methenamine and periodic acid thiosemicarbazide-osmium and are reactive with cations and with colloidal iron at pH 2.0, but become unreactive after methylation and saponification [53]. These results point to crystal ghosts in cartilage as pertaining to, or deriving from, acid proteoglycans, a conclusion strengthened by the observation that they react with CS-56, an antibody specific to the glycosaminoglycan portion of chondroitin sulfate [54]. Results reported by Appleton [55] and Davis *et al.* [56] also point to cartilage crystal ghosts as protein-carbohydrate complexes. Studies of early mantle dentin with soybean agglutinin-gold complexes [36] and with cetylpyridinium chloride-glutaraldehyde as a fixative [57] have shown filament- and needle-like structures similar to crystal ghosts reacting as proteoglycans. Similar results have been reported in bone [58] and in developing enamel [59].

Independently of their structure and composition, crystal ghosts are components of the organic matrix and must therefore pre-exist before the matrix begins to calcify. In this connection, the incubation of epiphyseal cartilage in lanthanum chloride shows in the still uncalcified matrix “focal filament aggregates”, which have a close resemblance with aggregates of crystal ghosts [60]. The reaction with lanthanum seems to reveal the same sites as those that react with calcium ions.

### 1.6. Supposed Function of Crystal Ghosts

The striking similarity between crystal ghosts and untreated early crystals leads to the supposition, first, that they are different morphological expressions of the same nanostructures or, in other words, that the early crystals are organic-inorganic, hybrid structures and, second, that their organic component functions as a reacting substrate that links the inorganic ions and behaves as a template: the filament-like shape of the crystals would then simply reflect the filament-like shape of the organic substrate. This shape is shared with the crystal ghosts of all vertebrate hard tissues; there are, however, ultrastructural differences (for instance, the crystal ghosts in enamel are much longer than those in bone and in cartilage), suggesting that molecules sharing the same general properties, but having a different composition, may be active in different tissues. The histochemical results show that crystal ghosts correspond to polymeric anionic molecules, which include the acid proteoglycans of epiphyseal cartilage, the phosphoproteins of dentin and the acidic glycoproteins of bone and enamel.

A number of reports have stressed the primary role that acidic molecules can play in the calcification of vertebrates and invertebrates [48,61,62,63,64,65,66,67,68]: they are generally thought to play a role in the nucleation process of hydroxyapatite (see [69]) and to control mineral formation and growth, not only through their high calcium ion binding capacity, but also by interacting with specific faces of crystals and by permitting or preventing their growth [70,71]. The finding of crystal ghosts as components of the early crystals suggests that their function is, rather (or in addition to), that of templates that link and stabilize mineral ions, giving rise to the early organic-inorganic structures and forcing them to acquire a filament-like shape. This would, incidentally, explain the observation of Beniash *et al.* [22] and Simmer *et al.* [24] (reported above) that the mineral morphology of enamel is determined prior to the formation of definitive crystalline structures. It also appears to be in line with the finding of Fang *et al.* [72] that the native and recombinant porcine amelogenins, P173 and rP172, stabilize initial mineral clusters and that, more importantly, rP172 regulates the organization of initial mineral clusters into linear chains. This suggested mechanism does not exclude other substances from playing a role in biocalcification: hydroxyapatite can be grown in gelatin [73], as well as in collagen type I fibrils [74]. Biomineralization is a complex process than may include complementary mechanisms.

**Figure 6:**
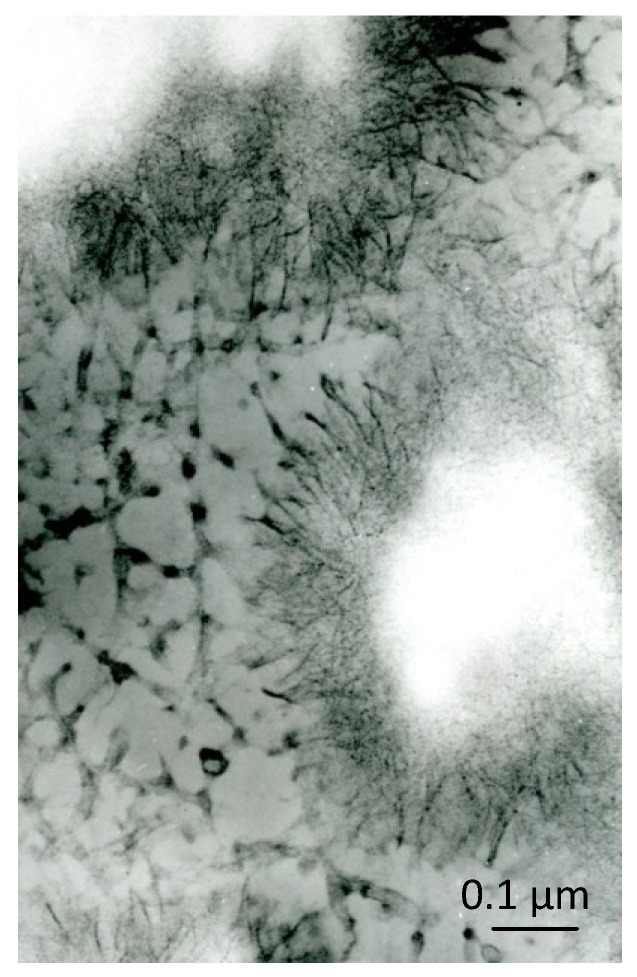
Cartilage calcification nodules after treatment with the PEDS method: crystal ghosts are recognizable at the border of the nodules, whose central zone appears empty. Formic acid-uranyl acetate and lead citrate, ×75,000. Scale bar: 7.5 mm = 0.1 µm.

The formation of the organic-inorganic, crystal-like, hybrid structures would function as an initial phase of the biocalcification process. As discussed above, a second, often neglected phase appears to follow, leading to the “maturation” of the crystals and to the removal of all crystal ghosts. This is documented by the fact that crystal ghosts become unrecognizable under the electron microscope as the degree of calcification rises: they do, in fact, gradually disappear from the central zone of the calcification nodules, where the calcification process is completed and is at its highest, final degree, and only remain recognizable at the periphery of the nodules, where the formation of crystals continues (Figure 6; see also [56]). At the same time, the Ca/P ratio increases in the central area of the nodules, and the electron diffractograms acquire the reflections and characteristics of poorly crystalline hydroxyapatite.

The disappearance of crystal ghosts implies their proteolysis. Actually, the loss of organic material has often been described during calcification. It typically occurs in enamel (reviewed by [75]), whose protein content falls from an initial value of 15%–20% by weight in immature enamel to 0.1% or less in the most mature enamel [76]. Proteolysis also occurs in bone, where a dramatic decrease in non-collagenous nitrogen takes place as incompletely calcified osteons reach the highest degree of calcification [77], and in epiphyseal cartilage, which loses about half of its content of proteoglycans [78] and 0.3% of sulfur [79] and whose proteoglycan aggregates undergo partial disaggregation and degradation and decrease in size during calcification [80,81,82,83].

The removal of the organic component from the early calcified particles may permit the amorphous inorganic substance, previously stabilized by crystal ghosts, to acquire an organized crystalline pattern.In this connection, it is of interest that a transient precursor phase has been described both in vertebrate and invertebrate hard tissues; this phase consists of amorphous calcium phosphate or carbonate and is stabilized by proteic molecules before becoming transformed into a crystalline phase [22,84,85,86,87,88,89]. *In vitro* surface-induced formation of apatite from simulated body fluid shows that the aggregation of prenucleation clusters leading to the nucleation of ACP precedes the development of oriented apatite crystals [90]. The removal of the organic stabilization molecules might be the factor that triggers the phase transformation. The proteolytic processing of P173 (full-length phosphorylated amelogenin) is required, for instance, to induce the transformation of amorphous calcium phosphate into apatitic enamel crystals, according to Kwak *et al.* [91].

## 2. Conclusions

An attempt to provide a definitive explanation of the mechanism of biological calcification would still be premature. An outline of the early phases of the calcification process should, in any case, include the crucial role of crystal ghosts as acidic organic molecules that link inorganic ions and give rise to organic-inorganic hybrids, whose organic constituent is subsequently removed, so allowing the inorganic component to acquire a crystalline organization.
